# Relationship between blood pressure level and activity of renin-aldosterone axis in patients with essential hypertension—a retrospective study

**DOI:** 10.7717/peerj.20883

**Published:** 2026-03-03

**Authors:** Peijuan Li, Bo Liao, Zhaoquan Yang, Jiulin Zhang, Kun Chen, Zhipeng Li

**Affiliations:** 1 General Practice Ward/International Medical Center Ward, General Practice Medical Center, West China Hospital, Sichuan University, Chengdu, China; 2 Department of Internal Medicine, Minzu Central Health Center, Haoba Town, Junlian County, Yibin, China; 3 General Department, Health Center, Jiangan Town, Jiangan County, Yibin, China

**Keywords:** Essential hypertension, Inappropriate hyperaldosteronemia, Diastolic blood pressure

## Abstract

**Background:**

A study indicates that elevated aldosterone levels increase the risk of hypertension by 16%. This retrospective cohort study examines the relationship between blood pressure and renin-aldosterone levels in patients with essential hypertension (EH), aiming to elucidate the clinical features and risk factors of inappropriate hyperaldosteronism in Chinese individuals with essential hypertension.

**Methods:**

Clinical data of 309 EH patients were analyzed. The participants were divided into three groups with a high (H)/medium (M)/low (L) blood pressure level: Group H (BP≥160 / (or) 100 mmHg, *n* = 151), Group M (BP 140–160/90–100 mmHg, *n* = 101), Group L (BP < 140/90 mmHg, *n* = 57). Since the data were all non-normal distributions, they were represented by the median and extreme values, and the Wilcoxon rank sum test was used. Categorical data were analyzed using the *χ*2 test. In the one-factor variance analysis, those with *P* < 0.05 were included in the multivariate analysis, and the multiple linear regression analysis method was used for the multivariate analysis. The study assessed the relationship between the plasma renin activity (PRA), plasma aldosterone concentration (PAC), and the aldosterone/renin activity ratio (ARR) in patients with EH and their blood pressure levels.

**Results:**

Among the 309 EH patients, 65 cases (21.04%) had elevated aldosterone levels, and 94 cases (30.42%) had increased renin activity. The diastolic blood pressure of patients with elevated aldosterone levels was higher than that of patients with normal or decreased aldosterone levels (100 *vs.* 95, 87, *p* < 0.05), while there was no significant difference in systolic blood pressure between patients with elevated aldosterone levels and those with normal or decreased aldosterone levels (152 *vs.* 151.5, 142, *p* > 0.05). The proportion of patients with heightened aldosterone levels was greater in Group H compared to the others (23.8% *vs.* 20.8% and 14.0%, *p* < 0.05). Multiple linear stepwise regression indicated that higher aldosterone levels correlated with increased diastolic blood pressure. PAC increased by 3.66 ng/dL for every 10 mmHg of DBP.

**Conclusion:**

The increase in aldosterone levels is relatively common among EH patients in China. The increase in aldosterone levels is correlated with the elevation of diastolic blood pressure. The elevated aldosterone levels are an independent risk factor for the increase in diastolic blood pressure in EH patients.

## Introduction

An epidemiological survey in China shows that the prevalence of hypertension in the adult population was as high as 25% (Yuming & Ning, 2015). Depending on the etiology, hypertension can be divided into essential hypertension (EH) and secondary hypertension. Primary aldosteronism (PA) is the most common cause of secondary hypertension, which is characterized by the overproduction of aldosterone by the adrenal gland. Aldosterone results in sodium and water retention *via* the mineralocorticoid receptor (Zennaro, Boulkroun & Fernandes-Rosa, 2015). Many studies have demonstrated that aldosterone excess exerts powerful effects on blood vessels and is associated with vascular stiffness and fibrosis of the kidneys, heart, and large arteries, and results in numerous deleterious consequences on the cardiovascular system (Funder et al., 2016; Buglioni et al., 2015; Fiebeler et al., 2007; Johar et al., 2006; Keidar et al., 2004; Qin et al., 2003; Korkmaz et al., 2000; Huan et al., 2012; Shen & Young, 2012). The aldosterone to renin ratio (ARR) is a recommended screening test by academic organizations and the ARR value 20–40 ng/dL: ng/ (ML h) is used as the cut-off value of a positive screening test (Funder et al., 2016). In clinical practice, we found that the serum aldosterone level was extraordinarily elevated in some EH patients even though the ARR was normal. It is not quite clear that there were any differences in characteristics of blood pressure (BP) in EH patients with and without inappropriate hyperaldosteronemia. Inappropriate hyperaldosteremia is refers to a non-renin-dependent increase in blood aldosterone levels that is above normal. Given that inappropriately elevated levels of aldosterone are believed to be a significant contributor to hypertension treatment resistance (Giacchetti et al., 2007; Velema et al., 2018; Williams et al., 2018), we should pay more attention to those EH patients with elevated aldosterone levels in clinical practice.

The aim of the study was to understand the clinical characteristics and related risk factors of EH patients with hyperaldosteronism.

## Materials and Methods

### Data sources and recruitment

We retrieved data from patients with hypertension who were referred to the West China Hospital between January 2012 and December 2017. Hypertension was defined as a systolic and diastolic BP greater than or equal to 130/80 mmHg according to the 2017 guideline of the American College of Cardiology/American Heart Association (Whelton et al., 2017). Plasma aldosterone concentration (PAC) and plasma renin activity (PRA) were tested for hypertensive patients who have drug-resistant hypertension, unexplained spontaneous or diuretic-induced hypokalemia, adrenal incidentaloma, sleep apnea, a family history of early-onset hypertension or a cerebrovascular accident at a young age (<40 y) (Funder et al., 2016), same as the ordinary hypertensive patients. They were measured at the moment of the clinical workup. The information was available for all eligible participants. PRA, PAC, and ARR were the exposures, and the outcomes were blood pressure levels of essential hypertension patients. The normal value reference ranges: PAC 9.8–27.5 ng/dl, PRA 0.93−6.56 ng/mL h.

Blood samples for PAC and PRA measurements were collected in the morning after the patient stayed in a sitting or standing position for at least 2 h and then was seated for 15 minutes. The aldosterone to renin activity ratio (ARR) was calculated as PAC (ng/dL)/PRA (ng/(mL h)). Both PRA and PAC were measured by radioimmunoassay. The commercial PRA kit was provided by Atomic Hi-Tech Co, Ltd, China. The intra- and inter-assay coefficients of variations for PRA were less than 10% and less than 15%, respectively. The commercial PAC kit was provided by Jiuding Medical Biological Engineering Co, LTD, China. The intra- and inter-assay coefficients of variation for PAC were 7.3% and 9.6%, respectively. Before the tests, the patients discontinued the use of potassium-wasting or -sparing diuretics and products derived from licorice root for at least 4 weeks and spironolactone for at least 6 weeks. Other drugs including β-blockers, angiotensin-converting enzyme inhibitors, and angiotensin receptor blockers were withdrawn for at least 2 weeks. Blood pressure was monitored after discontinuation. If blood pressure was higher than 160/90 mmHg, α-receptor antagonists (Prazosin or Terazosin tablets) were selected for blood pressure control, and non-dihydropyridine-calcium channel antagonists (verapamil sustained release tablets) were combined if the response was not satisfactory. Sodium intake was unrestricted. Patients with hypokalemia were provided adequate oral potassium supplements before the study.

The subjects included in this study were EH patients if they showed ARR values less than 30 (ng/dL)/(ng/mL h) on two occasions during clinical workup. Other inclusion criteria included age 20 ∼ 65 years old, urine analysis showed normal red blood cell and white blood cell counts, and no tubular type under high magnification field. Bilateral kidney size was normal in the ultrasound examination. The absence of renal artery stenosis was confirmed by Doppler ultrasound. Normal levels of plasma catecholamine, blood cortisol, urine-free cortisol, as well as normal serum creatinine and potassium levels were required. Patients who were older than 65 or with malignant tumors, and pregnant and lactating women were excluded.

This study was approved by the Ethics Committee of West China Hospital of Sichuan University and was granted exemption of informed consent (2021 Trial (No. 1320)).

### Data collection

We collected eligible subjects from the Biomedical Big Data Research Center of West China Hospital of Sichuan University. The clinical data and Lab results were recorded, including age, gender, height, weight, body mass index (BMI), course of hypertension, previous medical history and medication, blood, and urine routine, blood biochemistry, blood and urine cortisol, blood and urine catecholamine, abdominal color ultrasound, electrocardiogram, chest film, *etc.*

### Study design and measurements

In total, 309 participants were analyzed to determine the relationship between blood pressure level and activity of the renin-aldosterone axis in patients with essential hypertension. The participants were divided into three groups with high (H)/medium (M)/low (L) blood pressure level: Group H (BP≥160/(or) 100 mmHg), Group M (BP 140–160/90–100 mmHg), Group L (BP<140/90 mmHg). Then, we retrospectively analyzed the clinical data of the participants who had received the test for plasma renin activity and aldosterone concentration, and the relationship between blood pressure level and renin-aldosterone axis function.

### Statistical analyses

Statistical analysis was performed with SPSS statistical software. All evaluated parameters are expressed as the medians (25–75th percentiles) and extremum (*i.e.,* minimum to maximum) since the data are non-normal distributions. Wilcoxon rank sum test and *χ*2 test were used for classification data. Combined with professional knowledge and single-factor analysis of variance (*P* < 0.05), multi-factor analysis was carried out by using multiple linear regression analysis. Spearman rank correlation method was used to analyze the correlation among the indexes. A *p*-value of 0.05 was considered statistically significant.

## Results

### Clinical characteristics of the patients

A total of 309 patients with essential hypertension were enrolled in this study, including 175 males and 134 females, with a sex ratio of 1.3:1. The patient’s systolic blood pressure (SBP) was 150 mmHg, and the diastolic blood pressure (DBP) was 97 mmHg. The median renin activity was 3.22 ng/mL h (0.37–37.90 ng/mL h), the median aldosterone level was 20.0 ng/dL (7.38–83.7 ng/dL), and the median ARR was 6.18 ng/dL:ng/mL.h (1.0–28.19 ng/dL: ng/mL h).

### The blood pressure variation in patients with difference plasma renin activity and aldosterone concentration

A total of 309 hypertensive patients were divided into three groups according to the plasma renin activity and aldosterone concentration (Table 1). There were 65 patients (21.04%) with elevated aldosterone concentration (PAC > 27.5 ng/dL) and 94 patients (30.42%) with increased renin activity (PRA > 6.56 ng/mL h). There was no significant difference between groups in BMI (*P* > 0.05). The diastolic blood pressure was higher in the patients with elevated aldosterone concentration (*P* = 0.035) and increased renin activity (*P* < 0.001) when compared with that of the patients with normal aldosterone and normal renin activity.

**Table 1:** Plasma renin activity and aldosterone level distribution in patients with essential hypertension.

	Plasma renin activity (ng/mL h)				Plasma aldosterone level (ng/dL)			
	Increase	Normal	Decrease	*P* value	Increase	Normal	Decrease	*P* value
Number of people (%)	94 (30.4)	197 (63.4)	18 (6.2)	<0.001	65 (21.0)	238 (76.7)	6 (2.3)	<0.001
Male/Female	1.11	1.39	1.19	0.023	2.50	1.47	0.81	0.377
Age (year)	36 (17)	46 (19)	44.5 (19.75)	<0.001	35.5 (15.75)	45.5 (19)	37	0.002
Body mass index (kg/m^2^)	23.65 (5)	25.3 (5)	25.5 (6)	0.079	23.93 (6)	25.25 (5)	22.3	0.361
Systolic pressure (mmHg)	151.5 (31)	153 (27)	143.5 (20.25)	0.202	152 (29.25)	151.5 (26.75)	142	0.296
Diastolic pressure (mmHg)	99.5 (23.25)	95 (19)	89 (15.25)	<0.001	100 (26.25)	95 (19.75)	87	0.035

**Notes.**

The normal reference range: PRA: 0.93–6.56 ng/mL h; PAC: 9.8–27.5 ng/dl. PRA (increase group: PRA>6.56 ng/mL h, normal group: 6.56 ng/mL h ≥ PRA ≥ 0.93 ng/mL h, decrease group: PRA < 0.93 ng/mL h); PAC (increase group: PAC > 27.5 ng/dL, normal group: 27.5 ng/dL ≥ PAC ≥ 9.8 ng/dL, decrease group: PAC < 9.8 ng/dL). Since the data of age, BMI and pressure of the three groups were non-normally distribution. Median (interquartile distance) was used for descriptive statistics, and kruskal–Wallis H test was used for comparison between groups.

PRA plasma renin activity PAC plasma aldosterone concentration

### The variations in plasma renin activity and aldosterone level in patients with difference blood pressure level

According to the mean value of multiple blood pressure measurements, 309 patients were divided into three groups, H group (*n* = 151): BP≥160/(or) 100 mmHg; M group (*n* = 101): BP 140–160/90–100 mmHg; L group (*n* = 57): BP < 140/90 mmHg. There were no significant difference in gender, age, and BMI among the three groups (*P* > 0.05, Table 2). There was also no significant differences in serum creatinine, urea, and potassium levels among the three groups (*P* > 0.05). As shown in Table 3 the ratio of the patients with elevated aldosterone concentration and renin activity was higher in group H when compared with the other two groups (PAC: 23.8% *vs.* 20.8%*,* 14.0%; PRA: 35.8% *vs.* 23.8%, 28.1%).

**Table 2:** Comparison of general data of three groups of patients with hypertension.

	Group H	Group M	Group L	*P* value
Male/Female (case)	85/66	63/38	27/30	0.187
Age (yr. median (Min, Max))	42 (21, 65)	44 (21, 65)	48 (22, 65)	0.154
BMI (kg/m^2^, median (Min, Max))	25.2 (18.44, 43.34)	25.32 (15.82, 38.20)	24.60 (19.39, 32.00)	0.386

**Notes.**

Since the data of age and body mass index (BMI) of the three groups were non-normally distribution, Wilcoxon rank sum test was used. *χ*2 test was used to compare the gender differences between the two groups. Group H: BP ≥ 160/(or) 100 mmHg; Group M: BP 140–160/90–100 mmHg; Group L: BP < 140/90 mmHg.

BMI body mass index BP blood pressure

**Table 3:** Distribution of plasma renin activity and aldosterone level in three groups.

	Plasma renin activity(ng/mL h)			Plasma aldosterone level(ng/dL)		
	Increase (n)	Normal (n)	Decrease (n)	Increase (n)	Normal (n)	Decrease (n)
Group H (%)	54 (35.8)	94 (62.3)	3 (2.0)	36 (23.8)	113 (74.8)	2 (1.3)
Group M (%)	24 (23.8)	63 (62.4)	14 (13.9)	21 (20.8)	75 (74.3)	5 (5.0)
Group L (%)	16 (28.1)	40 (68.40	2 (3.5)	8 (14.0)	49 (86.0)	0 (0.0)

**Notes.**

Group H (*n* = 151): BP≥160/(or) 100 mmHg; Group M (*n* = 101): BP 140–160/90–100 mmHg; Group L (*n* = 57): BP < 140/90 mmHg.

BP blood pressure

The results showed that the *p*-value of PAC was less than 0.05 (*P* = 0.02), the PAC value of the three groups could be considered to be different; but the *p*-value of PRA and ARR were both greater than 0.05, the PRA value and ARR value of the three groups could not be considered to be different (Table 4).

**Table 4:** Comparison of plasma renin activity and aldosterone level in three groups.

	Group H	Group M	Group L	*P* value
PAC (ng/dL, median (Min, Max))	21.25 (7.97, 83.70)	18.61 (7.38, 74.20)	19.63 (9.99, 43.61)	0.020
PRA (ng/mL, median (Min,Max))	3.35 (0.89, 10.50)	3.05 (0.37, 37.90)	3.28 (0.44, 9.57)	0.211
ARR (median (Min, Max))	6.03 (1.27, 24.75)	5.92 (1.00, 28.19)	6.25 (1.53, 25.30)	0.523

**Notes.**

Since the data of PAC, PRA and ARR in three groups were non-normally distribution, Wilcoxon rank sum test was used. Group H: BP ≥ 160/(or) 100 mmHg; Group M: BP 140–160/90–100 mmHg; Group L: BP < 140/90 mmHg.

PRA plasma renin activity PAC plasma aldosterone concentration ARR aldosterone/renin activity ratio BP blood pressure

### The relationship between PAC, PRA, ARR and blood pressure level in patients with hypertension

PAC, PRA, and ARR were used as dependent variables, and the variables with correlation with PAC, PRA, and ARR were used as independent variables for multiple linear regression analysis (Table 5).

**Table 5:** Multiple linear regression analysis of PAC, PRA and ARR.

Dependent variable	Independent variable	Non standardization		Standardized regression coefficient	T value	*P* value	95% confidence interval
		Regression coefficient	Standard error				
**PAC**	Constant term	0.189	0.272		0.696	0.487	(−0.345∼0.723)
	Lg diastolic blood pressure	0.564	0.137	0.229	4.118	<0.001	(0.294∼0.834)
**PRA**	Constant term	−1.553	0.673		−2.306	0.022	(−2.879∼−0.227)
	Lg diastolic blood pressure	1.011	0.341	0.188	−2.966	0.003	(0.339∼1.782)
**ARR**	Constant term	2.585	0.635		4.072	<0.001	(1.335∼3.836)
	Lg diastolic blood pressure	−0.897	0.321	−0.171	−2.792	0.006	(−1.530∼−0.264)

**Notes.**

The data of PAC, PRA and ARR were non-normally distribution, the dependent variable is first transformed to logarithm, and the data after logarithm transformation is tested to be normal distribution, then multiple linear regression analysis is carried out. Stepwise regression method is used for analysis. The R-squared(R^2^) of these three models are 0.052, 0.035 and 0.029 respectively.

PAC plasma aldosterone concentration PRA plasma renin activity ARR aldosterone/renin activity ratio

In the multiple linear regression analysis of PAC, only diastolic blood pressure was included as a variable in the regression model. The multiple linear regression equation is LG (PAC) = 0.564 × LG diastolic pressure + 0.189. It can be seen that, after controlling for the potential influence of other variables, diastolic blood pressure is an independent and significant factor for predicting PAC. This means that when diastolic blood pressure increases by 10%, PAC increases by approximately 5.64%.

In the multiple linear regression analysis of PRA, only diastolic blood pressure was included as a variable in the regression model. The multiple linear regression equation is LG (PRA) = 1.011 × LG diastolic pressure – 1.553. It can be seen that after controlling for the potential influence of other variables, diastolic blood pressure is an independent and significant factor for predicting PRA. This means that when diastolic blood pressure increases by 10%, PRA increases by approximately 10.11%.

In the multiple linear regression analysis of ARR, only the diastolic blood pressure remained in the regression model. The multiple linear regression equation is LG (ARR) = (−0.897) × LG diastolic blood pressure + 2.585. It can be seen that, after controlling for the potential influence of other variables, diastolic blood pressure is an independent factor for predicting ARR. This means that when diastolic blood pressure increases by 10%, ARR decreases by approximately 8.97%.

Multiple linear regression analysis showed no significant correlation between SBP and PAC or PRA. No significant correlation was found between PAC and PRA.

## Discussion

Aldosterone, a hormone that induces the reabsorption of sodium, plays a fundamental role in salt and water homeostasis, blood pressure regulation, and cardiovascular remodeling. In normal physiological situations, aldosterone is regulated inversely with salt status. If aldosterone becomes inappropriately high for the salt status, elevated plasma aldosterone results in hypertension with subsequent potassium loss *via* the mineralocorticoid receptors, as seen in patients with primary aldosteronism.

Some clinical studies have found that inappropriately increased levels of aldosterone are also present in some patients with primary hypertension. In the Framingham Offspring Study, higher baseline serum aldosterone levels were independently associated with a 16% increased risk of elevation in BP and in 1688 normotensive patients there was a 17% greater likelihood of developing hypertension during a 4-year follow-up period (Vasan et al., 2004). Another study confirmed that individuals with hypertension had higher circulating aldosterone concentrations than those without hypertension (Cannone et al., 2018). The present study analyzes circulating aldosterone levels and their relationship with hypertension in 309 Chinese patients. The results showed that 21% of the EH patients had elevated plasma aldosterone levels and 30% had increased renin activity. The systolic and diastolic blood pressure was higher in patients with elevated aldosterone levels when compared with the patients with normal aldosterone concentrations. Our results confirmed that inappropriately increased aldosterone concentration is common in Chinese EH patients and hyperaldosteronemia may contribute to elevated blood pressure in some individuals with essential hypertension.

It is known that aldosterone not only maintains salt and water homeostasis but also has influences on vascular and cardiac remodeling because of its vasoconstrictor effect on blood vessels, by promoting a profibrotic environment and enhancing oxidative stress in the myocardium and blood vessels (Fiebeler et al., 2007; Johar et al., 2006; Keidar et al., 2004; Qin et al., 2003; Korkmaz et al., 2000). Thus, aldosterone promotes target organ damage involving the heart, kidneys, and brain independent of its blood pressure-elevating effects (Shen & Young, 2012; Giacchetti et al., 2007; Velema et al., 2018). In addition, the prevalence of the metabolic syndrome is reported to be higher in patients with primary hyperaldosteronism compared to patients with EH with a similar magnitude of blood pressure elevation (Buglioni et al., 2015), suggesting that aldosterone also has adverse metabolic consequences. In China, with the presence of high-sodium diets and increasing longevity, the harm of inappropriately elevated aldosterone in essential hypertensive patients should arouse the attention of clinicians.

Some studies have focused on the relationship between increased blood aldosterone levels and resistant hypertension (RHTN) in recent years (Funder et al., 2016; Sartori et al., 2006; Stowasser, 2014). RHTN is defined as blood pressure remaining above goal despite the concurrent use of 3 or more antihypertensive agents of different classes including a diuretic (Stowasser, 2014). Despite the various proposed pathophysiological mechanisms underlying RHTN, fluid overload is considered to be the main factor (Williams et al., 2018; Chobanian et al., 2003). A wide range of studies have demonstrated that fluid retention, mainly as a result of increased aldosterone concentration may play a key role in resistance hypertension (Calhoun et al., 2002). Our study found that the plasma aldosterone level in patients with blood pressure≥160/100 mmHg is significantly higher than in patients with blood pressure less than 160/100 mmHg. Even patients with BP between 140-160/90-100mmHg have higher aldosterone levels than those with slightly elevated blood pressure. The gender, age and BMI of the three groups showed no obvious difference, and regression analysis indicated that increased aldosterone level was an independent risk factor for elevated diastolic blood pressure. As the aldosterone level increases by 5.64%, the diastolic blood pressure increases by 10%. Therefore, our results suggest that elevated aldosterone level is not only related to resistant hypertension but is also associated with the degree of blood pressure in general hypertensive patients. The findings are consistent with another study (Buglioni et al., 2015). Our results confirmed that diastolic blood pressure was affected by PRA and PAC in patients with hypertension, while systolic blood pressure was not affected by PRA and PAC. We hypothesized that hyperaldosteronemia could cause the expansion of extracellular fluid volume, leading to a more significant increase in diastolic blood pressure. There were few other studies on the characteristics of diastolic blood pressure caused by elevated aldosterone levels. Mineralocorticoid receptor (MR) associated hypertension, which includes PA and RHTN, typically occurs in the context of elevated plasma aldosterone levels (Shibata & Itoh, 2012). Multiple studies have demonstrated that in EH patients with hyperaldosteronism, the addition of the mineralocorticoid receptor antagonist to standard therapy results in significant reductions in blood pressure and left ventricular volumes, supporting a relative volume overload state in these patients (Korkmaz et al., 2000; Williams et al., 2018; Tsutamoto et al., 2001; Paul et al., 2005; Rocha & Willams, 2002). Therefore, MR antagonists such as spironolactone would present as an effective treatment option to improve blood pressure control in the setting of aldosterone excess, by the diuretic effect and reductions in vascular stiffness.

This study was a retrospective study and lacked some important data, including urine sodium excretion, urine aldosterone excretion, and local population data on daily sodium intake. Further randomized controlled studies are needed to confirm these findings. The statistical analysis methods employed in this study had certain limitations, and the description of the screening process and biochemical operational procedures lacked sufficient detail. These aspects will be addressed and enhanced in future research designs.

## Conclusion

In summary, we reported the clinical characteristics of elevated blood aldosterone levels in patients with essential hypertension in China. It was found that the increase in aldosterone levels was relatively common among EH patients in China. The increase in aldosterone levels was correlated with the elevation of diastolic blood pressure. The elevated aldosterone levels were an independent risk factor for the increase in diastolic blood pressure in EH patients.

##  Supplemental Information

10.7717/peerj.20883/supp-1 Supplemental Information 1 Raw data

10.7717/peerj.20883/supp-2 Supplemental Information 2 Scatter plots illustrating the linear correlations between diastolic blood pressure and the logarithmic values of aldosterone and renin, respectively

## Additional Information and Declarations

### Data Availability

The following information was supplied regarding data availability: The raw measurements are available in the Supplemental File.
