# Supplementary material for: Relationship between blood pressure level and activity of renin-aldosterone axis in patients with essential hypertension—a retrospective study
Source: PeerJ. 2026 Mar 3;14:e20883. doi: 10.7717/peerj.20883 (PMC12965167; doi:10.7717/peerj.20883)
Supplement: Supplemental Information 2 [file peerj-14-20883-s002.pdf]

Scatter plot

1、Systolic blood pressure and Log values of PAC

ANOVA

	sum of squares	df	mean square	F	Sig.
Regression coefficients	.144	2	.072	2.508	.082
residual	15.620	545	.029		
total	15.764	547			

The independent variable is systolic blood pressure

coefficients

	Unstandardized coefficients		Standardized Coefficients	t	Sig.
	B	standard error	Beta		
SBP	.004	.004	.433	1.051	.294
SBP ** 2	-9.420E-6	.000	-.347	-.841	.401
（Constant）	.779	.275		2.827	.005

Log values of PAC

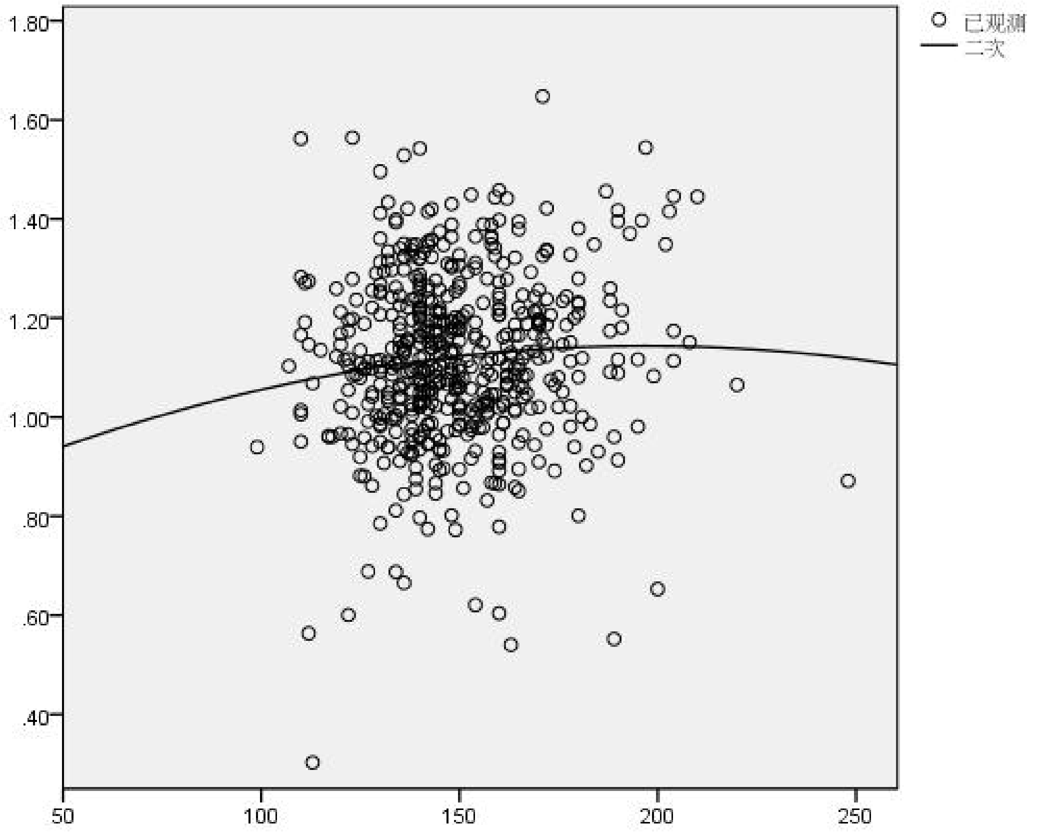

SBP

2、Diastolic blood pressure and Log values of PAC

ANOVA

	sum of squares	df	mean square	F	Sig.
Regression coefficients	1.251	2	.626	23.491	.000
residual	14.513	545	.027		
total	15.764	547			

The independent variable is diastolic blood pressure

coefficients

	Unstandardized coefficients		Standardized Coefficients	t	Sig.
	B	standard error	Beta		
DBP	.003	.004	.294	.843	.400
DBP ** 2	-6.792E-7	.000	-.012	-.034	.973
（Constant）	.821	.186		4.411	.000

Log values of PAC

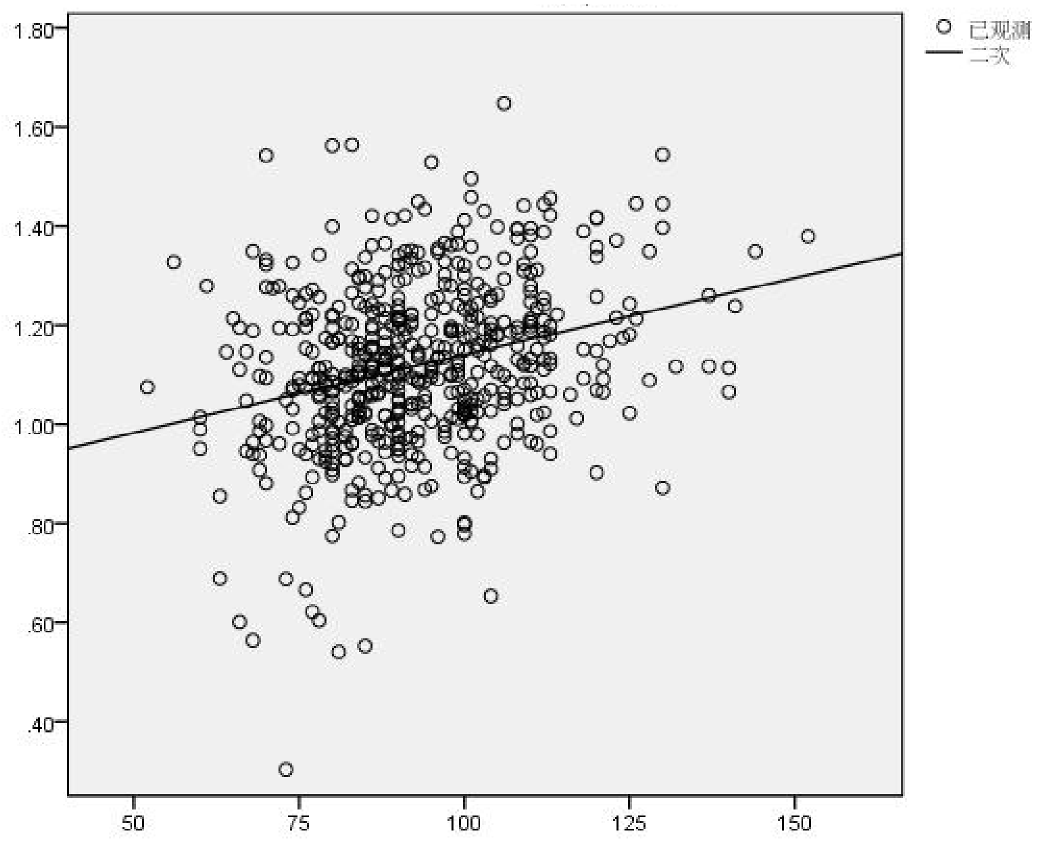

DBP

1、**Systolic blood pressure and Log values of** PRA

ANOVA

	sum of squares	df	mean square	F	Sig.
Regression coefficients	.538	2	.269	1.158	.315
residual	122.712	528	.232		
total	123.250	530			

The independent variable is systolic blood pressure

coefficients

	Unstandardized coefficients		Standardized Coefficients	t	Sig.
	B	standard error	Beta		
SBP	-.001	.010	-.046	-.110	.913
SBP ** 2	8.685E-6	.000	.111	.268	.789
（Constant）	-.138	.798		-.173	.862

Log values of PRA

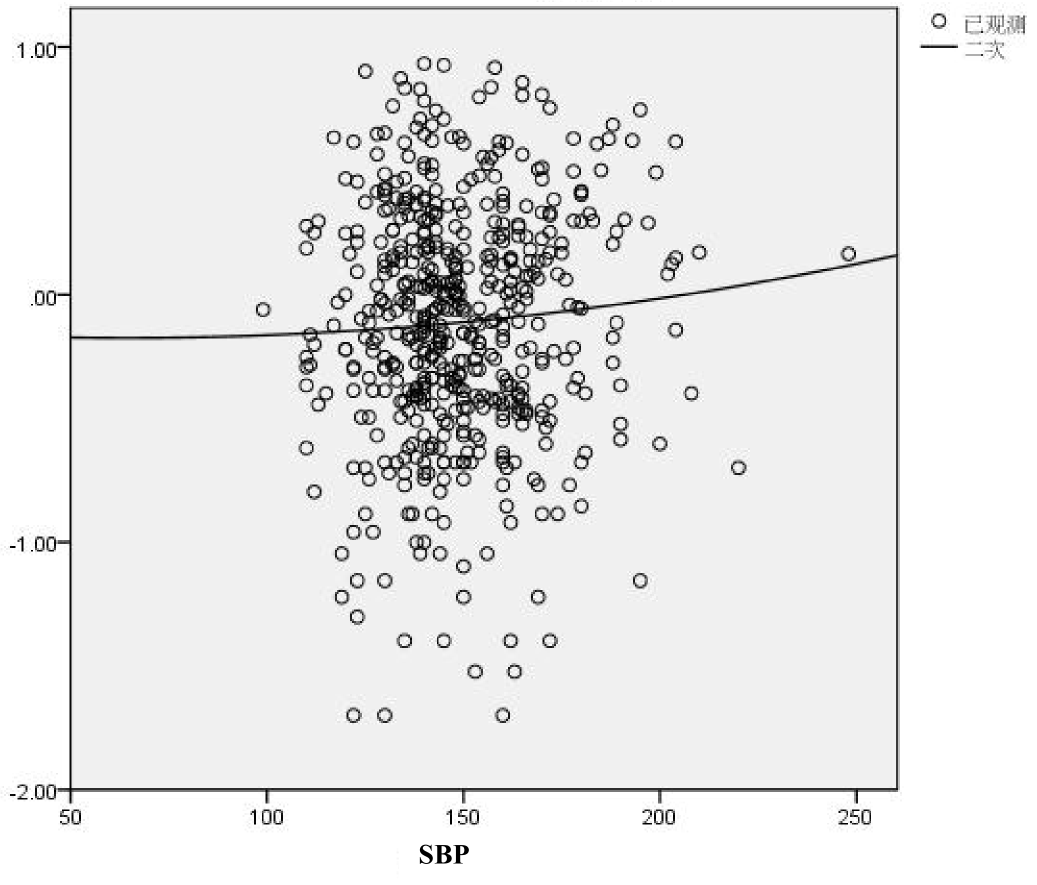

2、Diastolic blood pressure and Log values of PRA

ANOVA

	sum of squares	df	mean square	F	Sig.
Regression coefficients	8.884	2	4.442	20.508	.000
residual	114.366	528	.217		
total	123.250	530			

The independent variable is diastolic blood pressure

coefficients

	Unstandardized coefficients		Standardized Coefficients		
	B	standard error	Beta	t	Sig.
DBP	.008	.011	.255	.724	.469
DBP ** 2	2.280E-6	.000	.014	.040	.968
（Constant）	-.879	.536		-1.639	.102

Log values of PRA

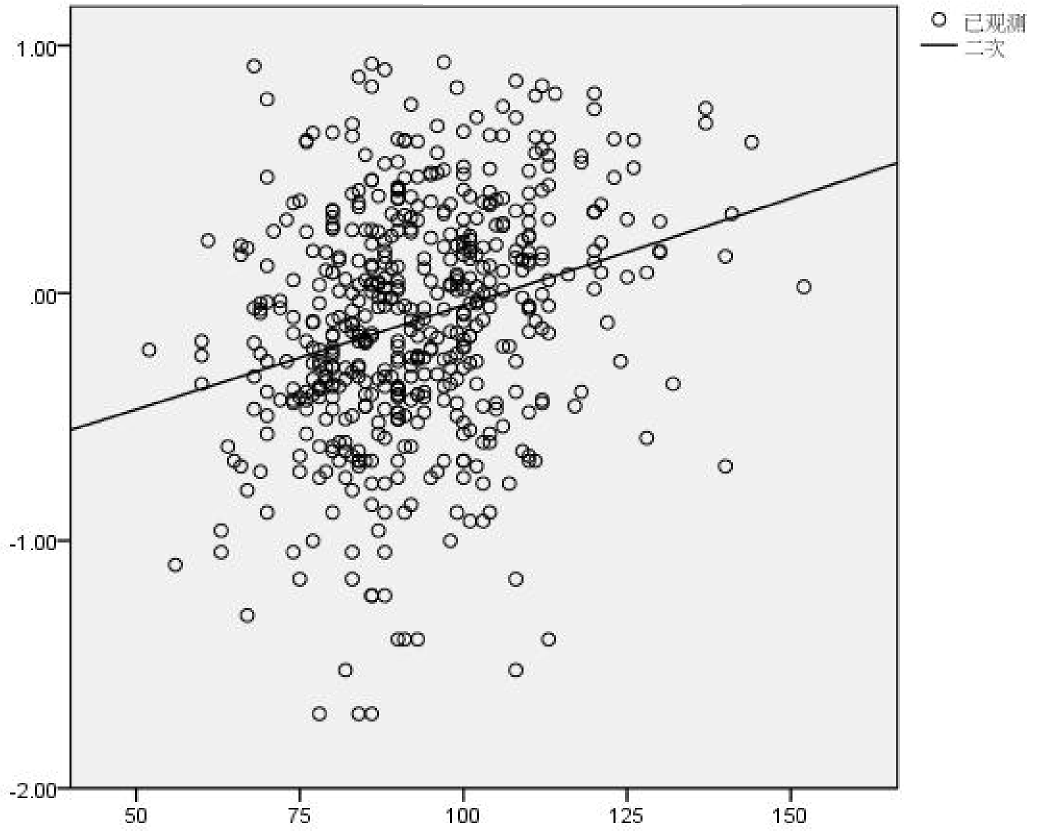

DBP

1、Systolic blood pressure and Log values of ARR

ANOVA

	sum of squares	df	mean square	F	Sig.
Regression coefficients	.945	2	.473	2.094	.124
residual	121.418	538	.226		
total	122.364	540			

The independent variable is systolic blood pressure

coefficients

	Unstandardized coefficients		Standardized Coefficients		
	B	standard error	Beta	t	Sig.
SBP	.010	.010	.430	1.038	.300
SBP ** 2	-3.873E-5	.000	-.504	-1.215	.225
（Constant）	.545	.784		.694	.488

Log values of ARR

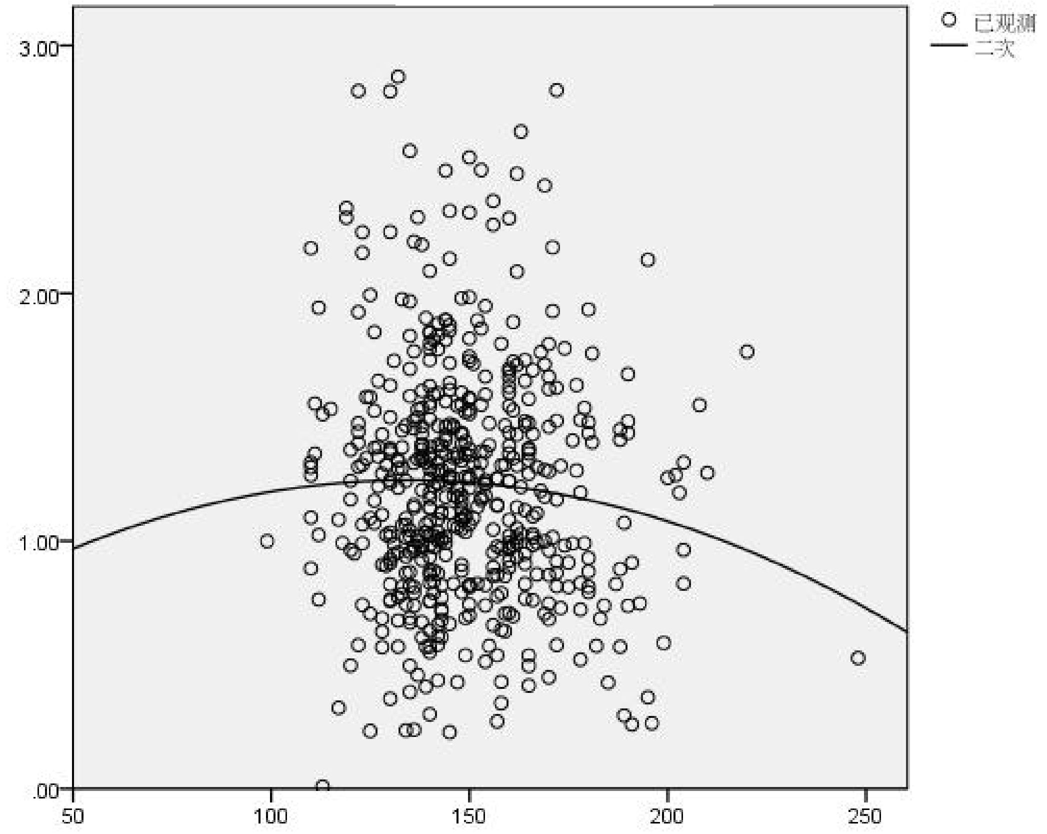

SBP

2、Diastolic blood pressure and Log values of ARR

ANOVA

	sum of squares	df	mean square	F	Sig.
Regression coefficients	5.978	2	2.989	13.816	.000
residual	116.386	538	.216		
total	122.364	540			

The independent variable is diastolic blood pressure

coefficients

	Unstandardized coefficients		Standardized Coefficients		
	B	standard error	Beta	t	Sig.
DBP	-.005	.011	-.157	-.442	.659
DBP ** 2	-1.039E-5	.000	-.065	-.182	.855
（Constant）	1.770	.534		3.316	.001

Log values of ARR

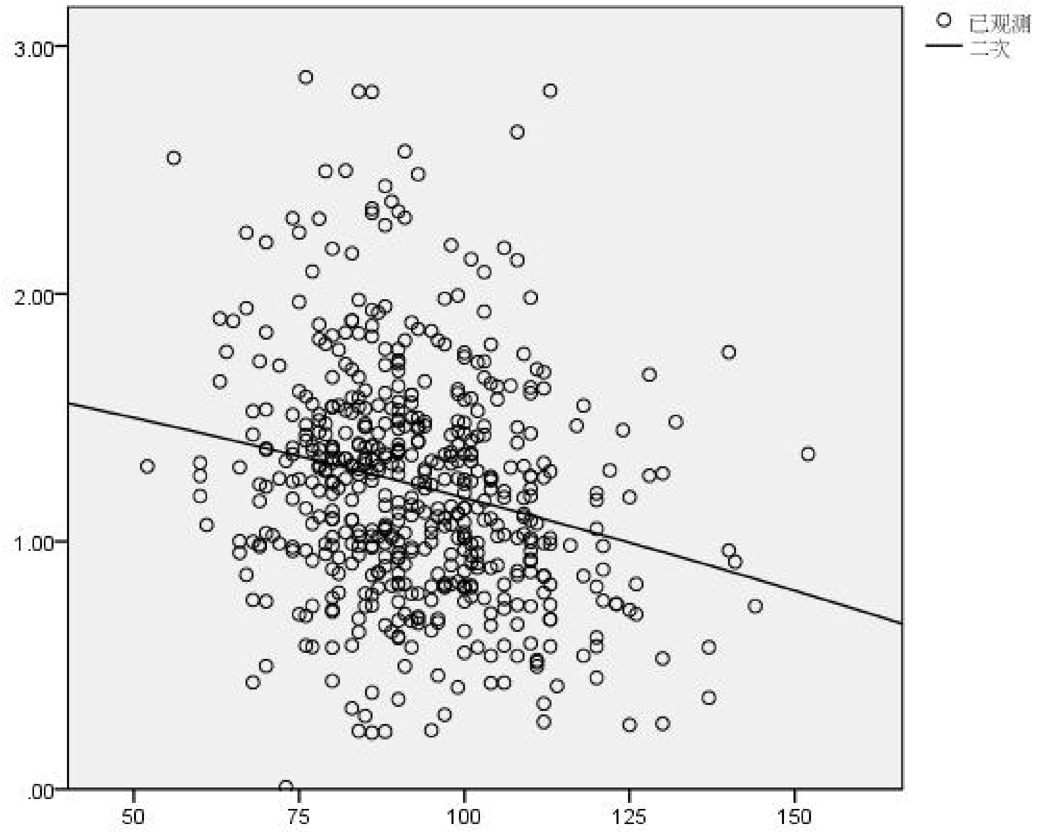

DBP
